# African-Lineage Zika Virus Replication Dynamics and Maternal-Fetal Interface Infection in Pregnant Rhesus Macaques

**DOI:** 10.1128/JVI.02220-20

**Published:** 2021-07-26

**Authors:** Chelsea M. Crooks, Andrea M. Weiler, Sierra L. Rybarczyk, Mason Bliss, Anna S. Jaeger, Megan E. Murphy, Heather A. Simmons, Andres Mejia, Michael K. Fritsch, Jennifer M. Hayes, Jens C. Eickhoff, Ann M. Mitzey, Elaina Razo, Katarina M. Braun, Elizabeth A. Brown, Keisuke Yamamoto, Phoenix M. Shepherd, Amber Possell, Kara Weaver, Kathleen M. Antony, Terry K. Morgan, Xiankun Zeng, Dawn M. Dudley, Eric Peterson, Nancy Schultz-Darken, David H. O’Connor, Emma L. Mohr, Thaddeus G. Golos, Matthew T. Aliota, Thomas C. Friedrich

**Affiliations:** a Department of Pathobiological Sciences, University of Wisconsin—Madison, Madison, Wisconsin, USA; b Wisconsin National Primate Research Center, University of Wisconsin—Madison, Madison, Wisconsin, USA; c Department of Veterinary and Biomedical Sciences, University of Minnesota, Twin Cities, St. Paul, Minnesota, USA; d Department of Comparative Biosciences, University of Wisconsin-—Madison, Madison, Wisconsin, USA; e Department of Pathology and Laboratory Medicine, University of Wisconsin—Madison, Madison, Wisconsin, USA; f Department of Biostatistics and Medical Informatics, University of Wisconsin—Madison, Madison, Wisconsin, USA; g Department of Pediatrics, University of Wisconsin—Madison, Madison, Wisconsin, USA; h Department of Obstetrics and Gynecology, University of Wisconsin—Madison, Madison, Wisconsin, USA; i Department of Pathology, Oregon Health and Science University, Portland, Oregon, USA; j Department of Obstetrics and Gynecology, Oregon Health and Science University, Portland, Oregon, USA; k U.S. Army Medical Research Institute of Infectious Diseases, Fort Detrick, Maryland, USA; University of North Carolina at Chapel Hill

**Keywords:** ZIKV, Zika virus, arbovirus, congenital Zika syndrome, flavivirus, macaque, pregnant

## Abstract

Following the Zika virus (ZIKV) outbreak in the Americas, ZIKV was causally associated with microcephaly and a range of neurological and developmental symptoms, termed congenital Zika syndrome (CZS). The viruses responsible for this outbreak belonged to the Asian lineage of ZIKV. However, *in vitro* and *in vivo* studies assessing the pathogenesis of African-lineage ZIKV demonstrated that African-lineage isolates often replicated to high titers and caused more-severe pathology than Asian-lineage isolates. To date, the pathogenesis of African-lineage ZIKV in a translational model, particularly during pregnancy, has not been rigorously characterized. Here, we infected four pregnant rhesus macaques with a low-passage-number strain of African-lineage ZIKV and compared its pathogenesis to those for a cohort of four pregnant rhesus macaques infected with an Asian-lineage isolate and a cohort of mock-inoculated controls. The viral replication kinetics for the two experimental groups were not significantly different, and both groups developed robust neutralizing antibody titers above levels considered to be protective. There was no evidence of significant fetal head growth restriction or gross fetal harm at delivery (1 to 1.5 weeks prior to full term) in either group. However, a significantly higher burden of ZIKV viral RNA (vRNA) was found in the maternal-fetal interface tissues of the macaques exposed to an African-lineage isolate. Our findings suggest that ZIKV of any genetic lineage poses a threat to pregnant individuals and their infants.

**IMPORTANCE** ZIKV was first identified in 1947 in Africa, but most of our knowledge of ZIKV is based on studies of the distinct Asian genetic lineage, which caused the outbreak in the Americas in 2015 to 2016. In its most recent update, the WHO stated that improved understanding of African-lineage ZIKV pathogenesis during pregnancy must be a priority. The recent detection of African-lineage isolates in Brazil underscores the need to understand the impact of these viruses. Here, we provide the first comprehensive assessment of African-lineage ZIKV infection during pregnancy in a translational nonhuman primate model. We show that African-lineage isolates replicate with kinetics similar to those of Asian-lineage isolates and can infect the placenta. However, there was no evidence of more-severe outcomes with African-lineage isolates. Our results highlight both the threat that African-lineage ZIKV poses to pregnant individuals and their infants and the need for epidemiological and translational *in vivo* studies with African-lineage ZIKV.

## INTRODUCTION

Zika virus (ZIKV) gained global notoriety in 2015, when it caused a large epidemic of febrile illness in the Americas and, for the first time, was causally associated with birth defects in infants born to mothers who became infected while pregnant (1). Why was ZIKV, which was first isolated in Uganda in 1947, not causally linked to birth defects prior to this outbreak in the Americas? Several hypotheses have emerged to explain why congenital ZIKV infection seems like a new complication, including the possibility that the unprecedented scale of the outbreak in the Americas revealed rare outcomes (2), that ZIKV infection clusters may not be detected, since the majority of infections are asymptomatic (2, 3), or that ZIKV circulating in the Americas acquired mutations that increased its ability to cause congenital ZIKV syndrome (CZS) (4).

ZIKV circulates as two genetic lineages, African and Asian. The vast majority of animal model and epidemiological studies of ZIKV to date have focused on Asian-lineage viruses, because they were responsible for the outbreak in the Americas. Therefore, relatively little is known about the pathogenic potential of African-linage viruses, particularly with regard to fetal outcomes. In cell culture experiments, African-lineage ZIKV isolates have been shown to replicate to higher titers and induce more cell lysis than Asian-lineage ZIKV (5–9). Particularly notable was the demonstration that African-lineage ZIKV isolates cause more-rapid and more-severe cytopathic effects (CPE) than Asian-lineage isolates in human embryonic stem cell-derived trophoblasts, which are critical for the development of the placenta (6, 7). Furthermore, in both pregnant and nonpregnant immunocompromised mouse models, African-lineage isolates have consistently shown both more mortality and more fetal harm than Asian-lineage strains (8–11). Several experiments have been conducted with African-lineage ZIKV isolates in nonpregnant macaques. Two of the isolates used in these studies have extensive passage histories in mice and therefore cannot be considered natural ZIKV isolates (9). Still, in one study, the virus replicated in the rhesus macaque host, but not as robustly as Asian-lineage isolates (12). A second study showed no replication of the African-lineage isolate in Mauritian cynomolgus macaques, while a third study showed replication comparable to that of Asian-lineage viruses and subsequent protection against heterologous challenge (13, 14). A low-passage-number isolate (Zika virus/Aedes africanus-tc/SEN/1984/41525-DAK) that has been used in several nonhuman primate models showed modest replication when inoculated intrarectally and intravaginally, and robust replication when inoculated subcutaneously; however, neither of these studies occurred during pregnancy (15, 16).

In the July 2019 epidemiological update on ZIKV, the WHO identified the assessment of fetal outcomes following infection with African-lineage viruses as a priority (17). This is underscored by the recent discovery of African-lineage ZIKV sequences in South America, including evidence of fetal harm in a nonhuman primate naturally exposed to an African strain of ZIKV most closely related to the type strain, MR766 (18, 19). While the ability of African-lineage ZIKV to infect the maternal-fetal interface and cause fetal harm has been rigorously studied in cell culture and immunocompromised mice, it remains unclear how translatable these findings are to humans.

To address this gap, we aimed to assess the pathogenic potential of a low-passage-number African-lineage ZIKV isolate during pregnancy in our well-established nonhuman primate model of ZIKV (20, 21). Recently, we demonstrated that the low-passage-number (5 cell culture passages), highly pathogenic African-lineage ZIKV strain ZIKV/Aedes africanus/SEN/DAK-AR-41524/1984 (ZIKV-DAK; BEI Resources, Manassas, VA) replicated to higher titers in maternal serum and caused significantly greater fetal harm than Asian-lineage ZIKV in pregnant *Ifnar1^−/−^* C57BL/6 mice (11). Notably, placental pathology was more severe in mice infected with ZIKV-DAK than in mice infected with an Asian-lineage virus. Since contemporary ZIKV isolates from Africa are not available through reagent repositories, this strain is one of the most recent low-passage-number isolates available for pathogenesis studies.

We infected four pregnant macaques with ZIKV-DAK during the late first trimester, monitored fetal health and growth throughout pregnancy, and assessed fetal outcomes (the presence of viral RNA [vRNA], gross abnormalities) at delivery at gestational day 155, approximately 1.5 weeks prior to full term. We compare data from a cohort of four pregnant macaques infected with ZIKV-DAK to data from a cohort of four pregnant macaques infected with Zika virus/H.sapiens-tc/PUR/2015/PRVABC59_v3c2 (ZIKV-PR), a low-passage-number Asian-lineage isolate. This virus, isolated from a human infected in Puerto Rico in 2015, has been well characterized in rhesus macaques (14, 20–24). Although we did not find evidence of more-severe fetal outcomes following infection with an African-lineage virus than with an Asian-lineage virus, the presence of a high burden of ZIKV vRNA in the placentas of ZIKV-DAK-infected macaques is concerning and suggests that African-lineage viruses may have a capacity to cause fetal harm similar to that of Asian-lineage viruses.

## RESULTS

### ZIKV-DAK replicates to high titers in macaques, with replication kinetics similar to those of ZIKV-PR.

**FIG 1:**
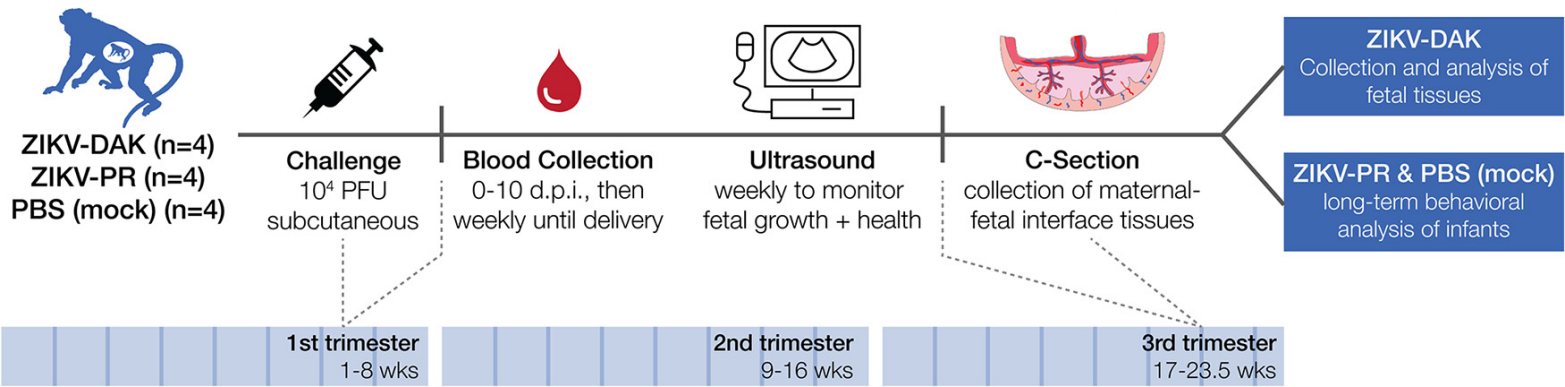
Study overview. Groups of four pregnant macaques were challenged between gestational days 45 and 50 (late first trimester) with either ZIKV-DAK, ZIKV-PR, or PBS (mock). Following viral challenge, blood was collected daily from 0 to 10 dpi, then twice weekly until viremia resolved, and then once weekly until delivery. Ultrasound was performed once weekly to measure fetal health and growth. Between gestational days 155 and 160 (1 to 1.5 weeks prior to full term), infants were delivered via cesarean section (C-section), and maternal-fetal interface tissues, including the placenta, fetal membranes, umbilical cord, and placental bed, were collected. Infants born to dams inoculated with ZIKV-DAK were humanely euthanized, and a comprehensive set of tissues was collected. Infants born to dams challenged with ZIKV-PR or PBS (mock) were paired with their mothers and followed for long-term behavioral analysis. Data from the long-term behavioral analysis will be published as part of a separate study.

**FIG 2:**
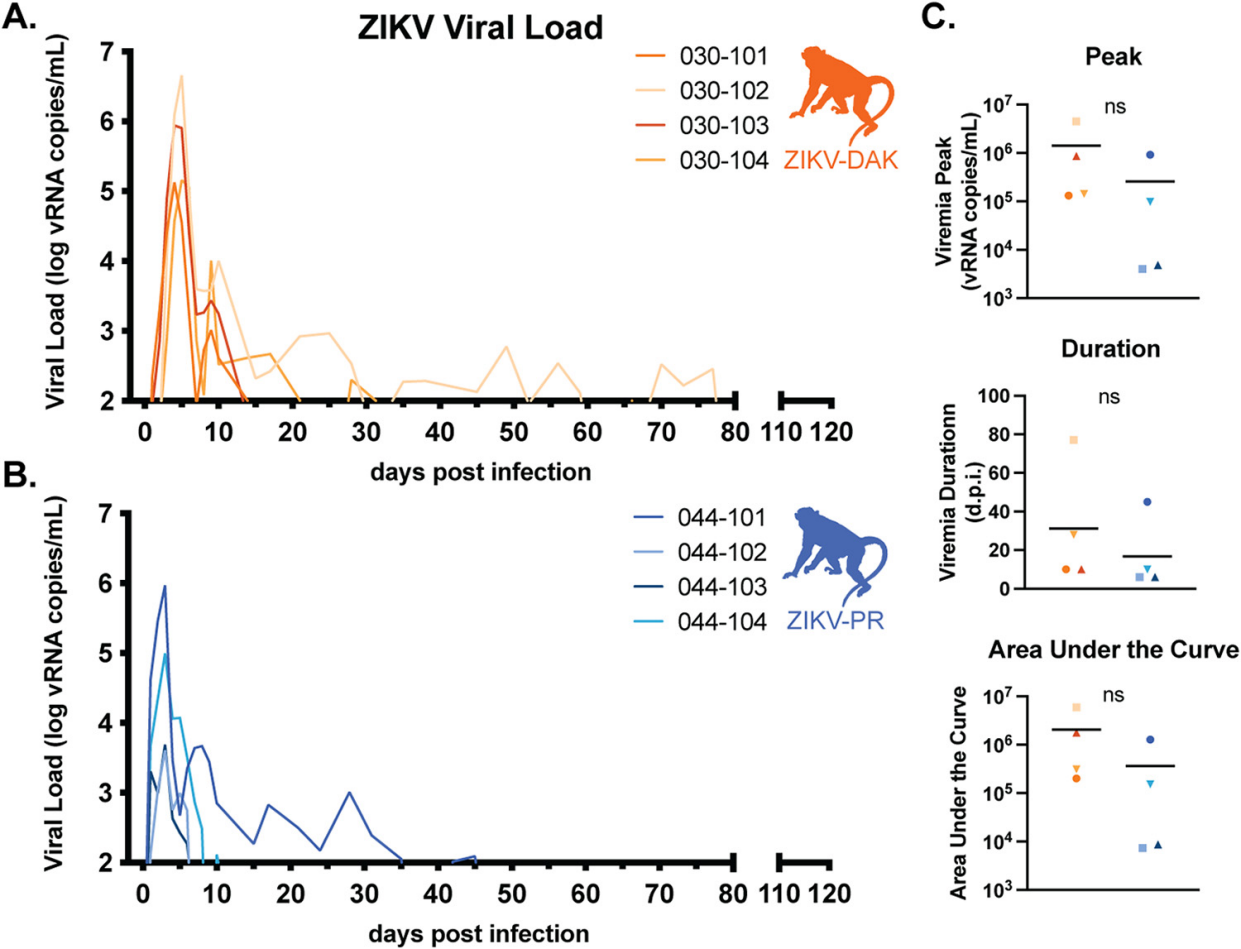
Replication kinetics of ZIKV-DAK and ZIKV-PR. (A and B) Viral loads were determined using ZIKV-specific QRT-PCR of RNA isolated from plasma. Only values above the assay’s limit of quantification (100 copies/ml) are shown. (C) There were no statistically significant differences in the peak, duration, or area under the curve of viremia between the two groups (by two-sample *t* tests).

Four pregnant rhesus macaques (Macaca mulatta) were subcutaneously inoculated with 10^4^ PFU of ZIKV-DAK between gestational days 45 and 50, late in the first trimester (Fig. 1). The first trimester is associated with the greatest risk of CZS in pregnant individuals and is both a time of active neurological development and a time at which many individuals do not yet know that they are pregnant (25, 26). Following inoculation, blood was collected daily for 10 days postinfection (dpi), then twice weekly until viremia resolved, and then once weekly for the remainder of gestation. Negative viremia was defined as two consecutive time points with plasma viral loads below the limit of quantification of our ZIKV quantitative reverse transcription-PCR (QRT-PCR) assay (100 copies/ml plasma). The virus replicated to high levels (10^5^ to 10^6^ vRNA copies/ml) in all four macaques, with viremia peaking between days 4 and 5 and persisting through day 10 for all four macaques; one macaque had prolonged viremia detected until 28 dpi and another until 77 dpi (Fig. 2A). In a cohort of four macaques infected with ZIKV-PR using the same inoculation and sampling regimen, viremia peaked on day 3 postinfection at 10^3^ to 10^5^ vRNA copies/ml. There were no statistically significant differences in viremia peak, duration, or area under the curve, suggesting that this African-lineage isolate replicated in pregnant macaques with kinetics similar to those of Asian-lineage isolates (Fig. 2C).

### ZIKV-DAK induces a robust nAb response.

**FIG 3:**
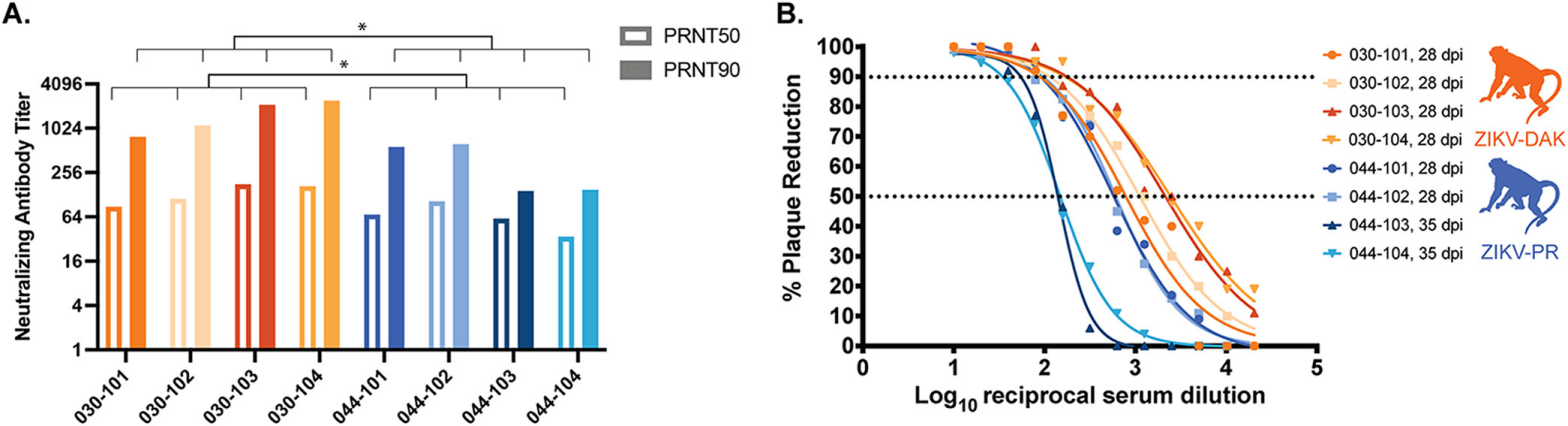
Neutralizing antibody titers. (A) Plaque reduction neutralization tests (PRNT) were performed on serum samples collected between 28 and 35 days postinfection to determine the titers of ZIKV-specific neutralizing antibodies. (B) Neutralization curves were constructed using GraphPad Prism. PRNT_90_ and PRNT_50_ values were estimated using nonlinear regression analysis and are shown on a bar graph (A) and indicated with dotted lines (B). PRNT_50_ and PRNT_90_ were compared using an unpaired parametric *t* test. ZIKV-DAK-infected macaques had significantly higher PRNT_50_ (*P* = 0.0371) and PRNT_90_ (*P* = 0.0243) than ZIVK-PR-infected macaques.

By 28 dpi, all macaques infected with either ZIKV-DAK or ZIKV-PR, regardless of viremia duration, had developed robust neutralizing antibody (nAb) titers (Fig. 3A). The 50% and 90% plaque reduction/neutralization titers (PRNT_50_ and PRNT_90_) developed in response to ZIKV-DAK infection were significantly higher than those developed in response to ZIKV-PR infection (Fig. 3B). The PRNT_90_ in macaques exposed to ZIKV-DAK were higher than the titers of macaques in a different study infected with the mouse-adapted isolate ZIKV-MR766 (1:20 to 1:40 at 21 dpi and 1:20 to 1:160 at 63 dpi), which were shown to be protective against heterologous challenge (14). Therefore, we expect that the immune response produced in these macaques infected with ZIKV-DAK during pregnancy would be protective against secondary ZIKV challenge.

### Regular ultrasound during gestation did not detect intrauterine growth restriction.

Sonographic imaging was conducted weekly beginning 1 week before inoculation to evaluate fetal health (heart rate), overall fetal growth (abdominal circumference [AC], femur length [FL]), and head growth (head circumference [HC], biparietal diameter [BPD]). No gross fetal or placental anomalies were observed. Various amounts of placental calcification were noted on ultrasound in all four macaques exposed to ZIKV-DAK; however, calcifications were also observed in macaques infected with ZIKV-PR and in mock-inoculated macaques, suggesting that these qualitative observations are normal for the gestational age or unrelated to viral infection.

**FIG 4:**
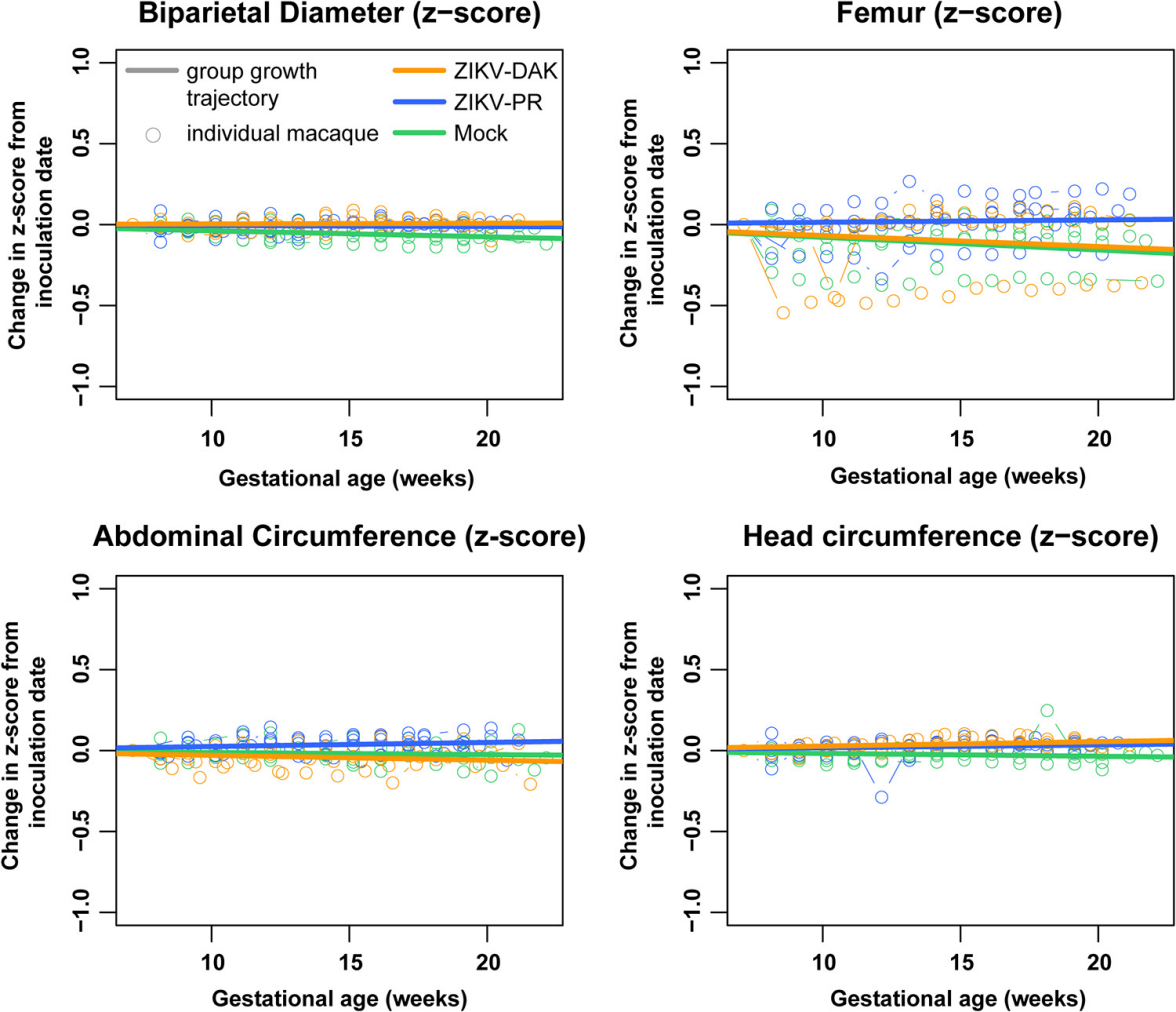
Intrauterine fetal growth. Sonographic imaging was performed weekly to measure fetal health and growth. Normative measurement data from the California National Primate Research Center were used to calculate Z-scores for each weekly measurement for each macaque. The change in the Z-score from the baseline measurement is plotted for each macaque with an open circle. Growth trajectories were quantified by calculating the regression slope parameters from baseline for each experimental group (solid lines) using a linear mixed-effects model with animal-specific random effects and an autoregressive correlation structure. Compared to the normative data, mock-inoculated animals had significantly reduced biparietal diameter growth (*P* = 0.0207); ZIKV-PR- and ZIKV-DAK-inoculated animals had very modest, but statistically significant, increases in head circumference growth (*P*, 0.0230 and 0.0179, respectively).

Femur length, abdominal circumference, head circumference, and biparietal diameter measurements were compared to normative data developed from 55 rhesus macaques at the California National Primate Research Center (27, 28). We calculated the number of standard deviations by which each fetal measurement differed from the normative data (Z-score) at that gestational age. A linear mixed-effects model with animal-specific random effects was used to evaluate the change in the outcome measures between gestational days 50 and 160. Growth was quantified by calculating the slope parameters for each experimental group. We then compared fetal growth in each group both to the normative data and to fetal growth in each of the other groups (Fig. 4). Compared to the normative data, mock-inoculated animals had significantly reduced biparietal diameter growth (*P* = 0.0207), while ZIKV-PR- and ZIKV-DAK-inoculated animals had very modest, but statistically significant, increases in head circumference growth (*P*, 0.0230 and 0.0179, respectively). All other values were not significantly different from the normative data. Importantly, when each of the experimental groups was compared to the mock-inoculated group, there was no significant reduction or increase in any of the growth measurements in the experimental group. This suggests that the few differences from the normative data that were observed may be due to differences between animal colonies and that infection with either lineage of ZIKV did not restrict or enhance fetal growth.

### No evidence of ZIKV vertical transmission was present at delivery.

At approximately gestational day 155 (full term is 165 ± 10 days in rhesus macaques), fetuses of ZIKV-DAK-infected dams were delivered via cesarean section (C-section) and humanely euthanized. No gross abnormalities were noted in any of the infants at delivery. A comprehensive set of maternal biopsy specimens, maternal-fetal interface tissues, and fetal tissues were collected for vRNA measurements and histopathological analysis. In the fetus, emphasis was placed on collecting tissues that may be involved in the transmission of the virus and tissues that are likely to be sites of ZIKV replication, including the central nervous system. Infants of ZIKV-PR-infected dams were delivered via cesarean section at approximately gestational day 160 and are being assessed for long-term neurodevelopmental sequelae. As a result, no fetal tissues were collected for comparison. No ZIKV RNA was detected in any of the fetal tissues collected from the four ZIKV-DAK pregnancies at the time of delivery (Table 1). Additionally, ZIKV IgM enzyme-linked immunosorbent assays (ELISAs) performed on serum samples from each of the infants born to dams infected with ZIKV-DAK were negative. Because pregnancies were allowed to go to near term, we cannot exclude the possibility that ZIKV-DAK was vertically transmitted earlier in gestation but cleared from the fetus before delivery. Histopathological examination of fetal tissues revealed evidence of minimal to mild neutrophilic lymphadenitis in 3 of 4 ZIKV-exposed animals. Because we also observed neutrophilic lymphadenitis in four mock-inoculated animals that underwent the same experimental regimen, this inflammation may be a feature of normal development or may have resulted from experimental procedures rather than viral infection.

**TABLE 1:** Maternal biopsy and fetal tissues

Tissue	No. positive/total tested	
	ZIKV-DAK	ZIKV-PR
Maternal		
Mesenteric lymph node	1/4	1/4
Spleen	2/4	N/A
Liver	0/4	N/A
Interplacental collateral vessels	N/A	0/4
Fetal		
Cerebrospinal fluid	0/4	N/A
Urine (aspirate)	0/4	0/1
Fetal plasma	0/4	0/1
Umbilical cord plasma	0/4	0/4
Amniotic fluid	0/4	0/4
Dura mater	0/4	N/A
Cervical spinal cord	0/4	N/A
Thoracic spinal cord	0/4	N/A
Lumbar spinal cord	0/4	N/A
Cerebrum, 1	0/4	N/A
Cerebrum, 3	0/4	N/A
Cerebrum, 4	0/4	N/A
Cerebrum, 6	0/4	N/A
Cerebrum, 7	0/4	N/A
Cerebrum, 9	0/4	N/A
Cerebrum, 10	0/3	N/A
Cerebellum, 1	0/4	N/A
Cerebellum, 2	0/4	N/A
Aqueous humor (aspirate)	0/4	N/A
Optic nerve	0/4	N/A
Sclera	0/4	N/A
Cornea	0/4	N/A
Retina	0/4	N/A
Pericardium	0/4	N/A
Heart full thickness section	0/4	N/A
Aorta, thoracic	0/4	N/A
Lung	0/4	N/A
Uterus or seminal vesicle	0/4	N/A
Testes or ovary	0/4	N/A
Adipose tissue, omentum	0/4	N/A
Epidermis/dermis of abdomen	0/4	N/A
Muscle, quadriceps	0/4	N/A
Bone marrow	0/4	N/A
Oropharyngeal lymph node, tonsil	0/4	N/A
Spleen	0/4	N/A
Thymus	0/4	N/A
Submandibular lymph node	0/4	N/A
Tracheobronchial lymph node	0/4	N/A
Mesenteric lymph node	0/4	N/A
Axillary lymph node	0/4	N/A
Inguinal lymph node	0/4	N/A
Esophagus	0/4	N/A
Stomach	0/4	N/A
Duodenum	0/4	N/A
Jejunum	0/4	N/A
Ileum	0/4	N/A
Cecum	0/4	N/A
Colon	0/4	N/A
Liver	0/4	N/A
Tongue	0/4	N/A
Urinary bladder	0/4	N/A
Kidney	0/4	N/A
Spleen	0/4	N/A
Thyroid	0/4	N/A
Adrenal gland	0/4	N/A
Pancreas	0/4	N/A

### ZIKV is present in a variety of maternal-fetal interface tissues at delivery.

Macaques typically have a bidiscoid placenta (29). To understand ZIKV distribution in the placenta, each placental disc was dissected into its individual cotyledons (perfusion domains), and samples from the decidua, chorionic villi, and chorionic plate were taken from each cotyledon for both viral loads and histology (29). To understand ZIKV distribution in the maternal-fetal interface, additional samples were taken from the fetal membranes, uterine placental bed, and umbilical cord for both viral loads and histology. To assess the presence of virus in the dam, biopsy samples of the mesenteric lymph node, liver, and spleen were taken from dams exposed to ZIKV-DAK for viral loads and histology. Only mesenteric lymph node biopsy specimens were collected from dams exposed to ZIKV-PR. In the ZIKV-DAK dams, 3 of 12 biopsy specimens, representing 2 different tissue types from 2 different macaques, were positive (Table 1). In the ZIKV-PR dams, 1 of 4 mesenteric lymph node biopsy specimens was positive.

**FIG 5:**
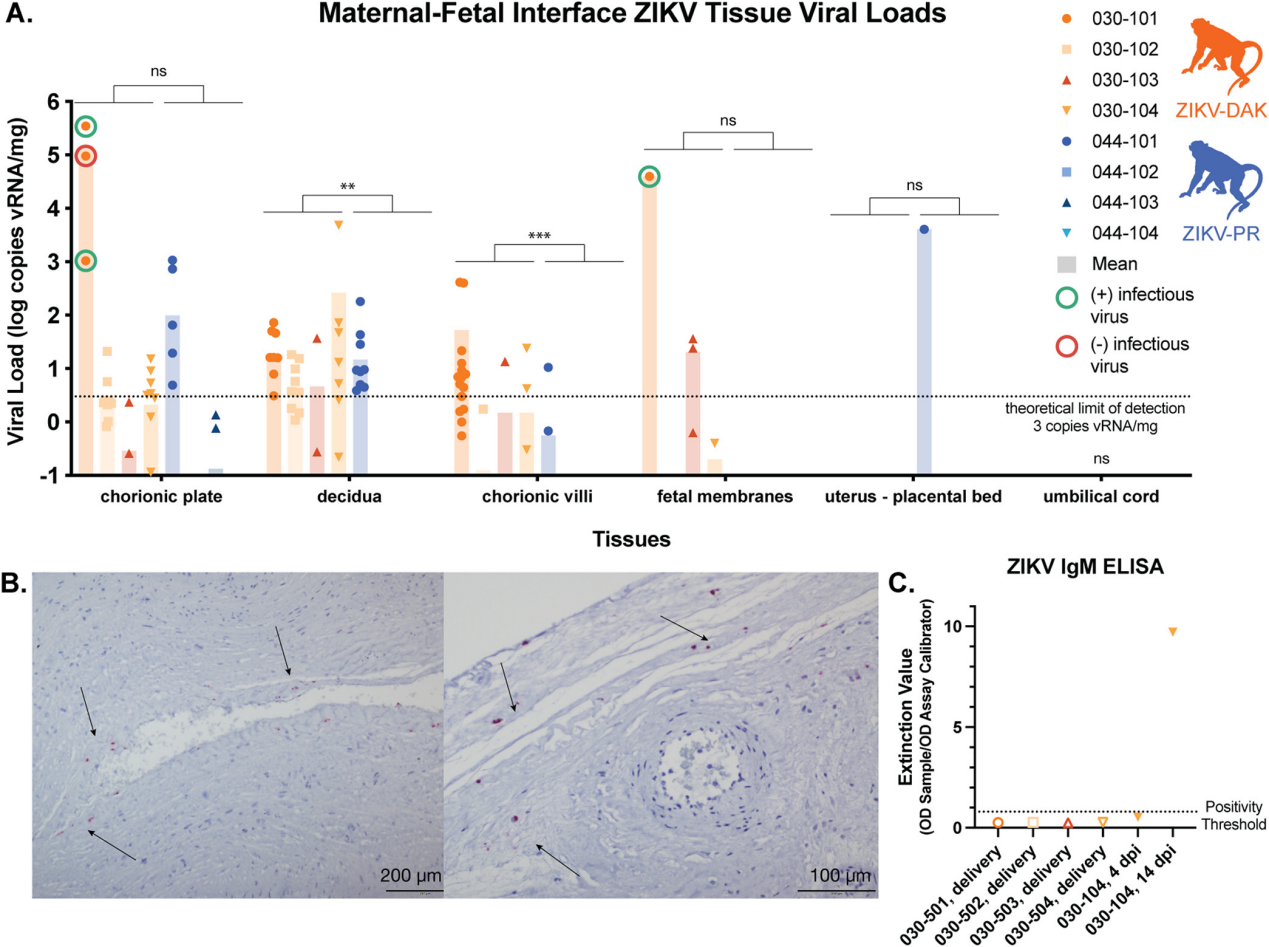
vRNA at the maternal-fetal interface. For each macaque, tissue biopsy specimens were collected from the chorionic plate, chorionic villi, and decidua from each placental cotyledon; one to three biopsy specimens were collected from the fetal membranes; and one biopsy specimen was collected from the uterine placental bed and umbilical cord. Viral loads were determined by ZIKV-specific QRT-PCR from RNA isolated from tissue samples. (A) Viral loads of maternal-fetal interface tissues. A nonparametric Mann-Whitney test was used to assess the statistical significance of differences between the experimental groups in samples containing more than the theoretical limit of detection of 3 copies vRNA/mg tissue (**, *P* < 0.01, ***, *P* < 0.001; ns, not significant). (B) Representative images from *in situ* hybridization performed on fixed tissue sections from each of the placental cotyledons from macaque 030-101. Positive staining for ZIKV RNA (red; indicated by arrows) was identified in 11 of the 17 cotyledons tested, primarily in the chorionic plate. (C) Serum samples from the four infants born to ZIKV-DAK-infected mothers were tested via ELISA for the presence of ZIKV IgM antibodies. Serum samples from dam 030-104 were included as positive (14 dpi) and negative (4 dpi) controls.

ZIKV RNA was present in the placenta and maternal-fetal interface in all four ZIKV-DAK-infected animals to various degrees regardless of the duration of viremia (Fig. 5A). The highest burdens were found in the decidua (basalis), chorionic plate, and chorionic villi; lower levels were found in the fetal membranes. No vRNA was identified in the placental bed of the uterus, in umbilical cord tissues (Fig. 5A), or in the amniotic fluid or umbilical cord blood (Table 1). In contrast, fewer maternal-fetal interface tissues of macaques infected with ZIKV-PR were positive. All but one of the tissues positive for ZIKV-PR RNA were from a single macaque, 044-101. No vRNA was detected in the umbilical cord (Fig. 5A), amniotic fluid, or umbilical cord plasma (Table 1) in ZIKV-PR-infected animals. Compared to those of the cohort of macaques infected with ZIKV-PR, significantly greater burdens of ZIKV vRNA are present in the deciduae, chorionic plates, and chorionic villi of the ZIKV-DAK cohort (Fig. 5A). There were no significant differences in vRNA burdens in the fetal membranes, uterine placental bed, or umbilical cord.

To assess whether there was replicating virus in the placenta at delivery (105 to 113 days postinfection), three high-viral-load (>10^3^ copies/mg) chorionic-plate samples and one fetal-membrane sample from macaque 030-101 were tested in a plaque assay. Of these four tissues, two chorionic plates and one fetal membrane were positive for infectious virus (Table 2). To further understand the distribution of vRNA within the placenta, tissue sections of the placental cotyledons from macaque 030-101 were evaluated using *in situ* hybridization (ISH). ISH probes for the ZIKV genome were used to identify ZIKV RNA in the tissue sections. Eleven of the 17 cotyledons tested were positive for ZIKV RNA, primarily in the chorionic plate, which is consistent with both the QRT-PCR and the plaque assay results (Fig. 5B).

**TABLE 2:** Infectious virus in maternal-fetal interface tissues

Tissue	Viral load (vRNA copies/g)	Viral titer (PFU/g)
Chorionic plate 1	1.53E+06	3.93E+01
Chorionic plate 2	6.47E+08	4.69E+02
Chorionic plate 3	1.41E+08	0
Fetal membranes	6.55E+07	2.55E+01

### Comprehensive histological examination of placental tissues.

**FIG 6:**
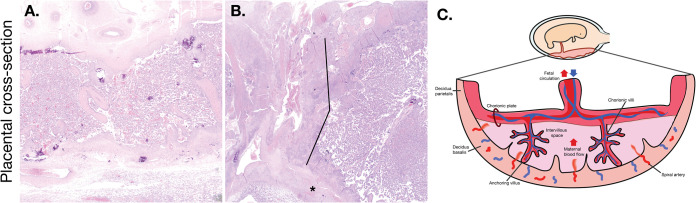
Representative images of ZIKV-DAK placental pathology. (A) Normal placental cross section. The decidua basalis is located at the bottom of the cross section; the chorionic plate is located at the top. The placental parenchyma has a spongy appearance as a result of a robust network of villi. (B) Transmural infarction was noted in 4 of 4 macaques infected with ZIKV-DAK. Lines indicate the region of infarcted tissue (area of nonfunctional, ischemic, villous parenchyma), and the asterisk indicates the trophoblastic shell of the basal plate. (C) Graphical representation of a cross section of the placenta. The orientation of the diagram corresponds to the orientation of the cross sections in panels A and B.

**FIG 7:**
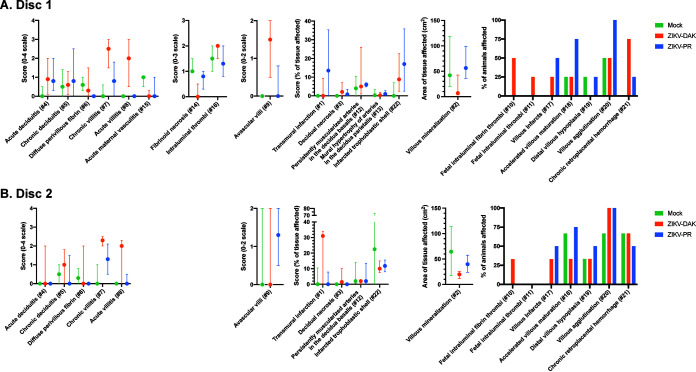
Placental pathology scoring. The central cross section of each placental disc was evaluated for 22 pathological features. Some features were specific to the fetal membranes or uterus; these are found only under the disc 1 scoring. Statistical pairwise comparisons between groups were performed for each feature. For quantitative features (features 1 to 9, 12 to 16, and 22), a nonparametric Wilcoxon rank sum test was used; for binary features (features 10, 11, and 17 to 21), Fisher’s exact test was used. For quantitative features, the median value is shown, with error bars representing the interquartile range. Pairwise comparisons revealed no statistically significant differences for any of the features.

To better understand the impact of *in utero* ZIKV-DAK infection, maternal-fetal interface tissues were evaluated microscopically. Gross histopathological evaluation of the maternal-fetal interface tissues of ZIKV-DAK-exposed animals revealed a primary qualitative finding of transmural infarction of the central section of the placenta (Fig. 6). Transmural placental infarctions are areas of ischemic necrotic placental villi extending from the trophoblastic shell of the basal plate to the chorionic plate and are considered to be a result of a lack of oxygenated maternal blood flow (30). These infarctions were present in all four macaques infected with ZIKV-DAK. In contrast, they were observed in 2 of 4 macaques infected with ZIKV-PR and 1 of 4 mock-inoculated macaques. In order to quantitatively assess the pathologies present in the maternal-fetal interface, a central cross section of each placental disc was scored for 22 functional features (Fig. 7). There were no statistically significant differences between either of the experimental groups and the mock-inoculated controls for any of the scored features; however, there was a trend toward increased chronic and acute villitis in the ZIKV-DAK-exposed animals.

Further histological analysis examined a cross section of each of the individual placental cotyledons for the presence of chronic histiocytic intervillositis (CHIV), infarctions, villous stromal calcifications, and vasculopathy (Table 3). We also compared placental weights. There were no statistically significant differences in weight or pathological findings between the experimental and control groups for any of the features. We were somewhat surprised by the finding of infarction in mock-inoculated controls in this study. The presence of infarctions, not often observed in mock-inoculated controls, could indicate that the pathology observed in this analysis is a result of normal placental maturation. The cross sections observed in this analysis included tissue from the placental periphery, where placental infarctions are normal at term. Additionally, the mock-inoculated controls underwent the same weekly anesthesia and experimental procedures as the experimental cohorts. Stress from experimental procedures and anesthesia may have resulted in infarctions, which underscores the need to include mock-inoculated controls when one is evaluating tissues for viral pathogenesis. Although initial qualitative examination of the central section of the placental disc suggested that there was increased placental pathology in the ZIKV-DAK cohort, a more-detailed analysis of the central sections of both the whole disc and individual cotyledons suggests that the increased vRNA burdens in the placentas of ZIKV-DAK macaques did not lead to significantly enhanced placental pathology.

**TABLE 3:** Placental cotyledon pathology

Group	Dam	% of cotyledons:		Presence or absence of:		Placental wt (g)
		CHIV^+^	Infarcted	Villous stromal calcifications	Vasculopathy	
Mock	044-105	0.0	5.88	Present	Absent	111.08
	044-106	0.0	12.5	Present	Absent	106.5
	044-107	0.0	0.0	Present	Present	144.48
	044-108	0.0	45.5	Present	Absent	122.92
ZIKV-DAK	030-101	0.0	43.8	Absent	Absent	131.54
	030-102	0.0	0.0	Absent	Absent	111.4
	030-103	0.0	66.7	Absent	Present	135.32
	030-104	0.0	57.9	Present	Present	124.71
ZIKV-PR	044-101	0.0	25.0	Present	Absent	172.59
	044-102	0.0	33.3	Present	Absent	123.87
	044-103	0.0	0.0	Absent	Absent	134.49
	044-104	0.0	18.2	Absent	Absent	120.48

## DISCUSSION

Here, we provide the first comprehensive analysis of a low-passage-number African-lineage ZIKV isolate in pregnant nonhuman primates. The data presented here demonstrate that this African-lineage ZIKV isolate is capable of robust replication in rhesus macaques. Infection induces a strong neutralizing antibody response, at or above titers that have been shown to be protective against challenge 2 years following primary challenge (31). Regular monitoring of fetal growth via ultrasound did not reveal any significant intrauterine growth restriction compared to the growth of mock-inoculated animals. ZIKV infection of the placenta has been shown to be focal (23); therefore, in addition to assessment of well-established sequelae of viral infection at the maternal-fetal interface, we completed an extensive virological and histological evaluation of the placenta at delivery. Viral load testing of tissues from the extensive dissection of the placental discs into individual cotyledons and specific segments thereof revealed significantly higher burdens of ZIKV in the decidua and chorionic villi in animals exposed to ZIKV-DAK. Although the vRNA burdens in the chorionic plates and fetal membranes of the ZIKV-DAK cohort were not significantly higher than those in ZIKV-PR animals, macaque 030-101 (ZIKV-DAK) had particularly high vRNA burdens in the chorionic plate and fetal membranes. Plaque assays confirmed the presence of infectious virus in these tissues, and ISH confirmed a high vRNA burden in the chorionic plate. Despite the presence of ZIKV in the chorionic plate—the fetal side of the placenta—including infectious virus detected in one animal, there was no evidence of vertical transmission at delivery in either group. Unfortunately, current diagnostic testing remains suboptimal and inconsistent for the detection of congenital ZIKV infection in humans, and these data suggest that the vRNA burden in the maternal-fetal interface is not a robust predictor of clinical outcome for the fetus at birth This does not, however, preclude the possibility that infants may develop clinical sequelae later in life due to viral exposure and/or placental insufficiency during gestation. Many normal-appearing infants exposed to ZIKV *in utero* develop neurodevelopmental delays in the years after birth (32–35).

This cohort of macaques infected with an African-lineage virus was compared to a cohort of macaques infected with an Asian-lineage virus and a mock-inoculated control group. Based on previous studies in cell culture and mice, we expected to see a more-severe phenotype in the macaques that were infected with the African-lineage virus (5–11). We expected this more-severe phenotype to manifest as enhanced viral replication (as determined by a higher peak or longer duration of viremia), gross fetal abnormalities at delivery, or fetal demise. However, the only feature that differed significantly between the experimental groups was an increase in the vRNA burdens in the chorionic villi and decidua.

To date, few studies of Asian-lineage viruses in nonhuman primates have shown clear evidence of fetal harm, despite a clear causal association between Asian-lineage ZIKV and CZS. A minority of human pregnancies known to be affected by ZIKV result in CZS (5 to 14%) or fetal loss (4 to 7%) (2); therefore, it is perhaps unsurprising that there is limited evidence of fetal harm in small studies with nonhuman primates. In this study, ZIKV-DAK infection of pregnant macaques resembled ZIKV-PR infection across several parameters, including infection of maternal-fetal interface tissues. Unfortunately, the absence of ZIKV RNA in ZIKV-DAK-exposed fetuses prevented us from rigorously establishing the likelihood for vertical transmission by this strain, so this experiment, limited by the small number of animals used in nonhuman-primate studies, could not conclusively resolve questions related to the potential of ZIKV-DAK to cause fetal harm. The inability to detect ZIKV RNA in affected fetal tissues could be due to the focal nature of infection, assay sensitivity, and/or viral clearance by the time of necropsy, which falls 105 to 113 days after the infection of the dam. Although we did not observe direct fetal harm, the presence of a high burden of ZIKV vRNA in the placenta is concerning and suggests that African-lineage viruses may have a capacity to cause fetal harm similar to that of Asian-lineage viruses. African-lineage ZIKV should be considered a threat to pregnant individuals and their infants, which should be taken into account when one is providing public health guidance. While African-lineage ZIKV had been thought to be geographically confined to Africa, recent studies have identified sequences of African-lineage ZIKV in South America (18, 19). This highlights the need for continuing study of ZIKV of both genetic lineages.

A significant limitation of this study is the small sample size (*n* = 4) in each of the experimental groups. Particularly in studying a pathogen whose most-severe effects are found only in a minority of cases (2), modeling rare events in a small study is difficult, and we cannot capture the full range of disease experienced by people infected with ZIKV during pregnancy. We also tested a single inoculation dose, virus strain, and inoculation time point; different experimental conditions may reveal different outcomes, which could include starker differences between the lineages. Furthermore, this study focused on characterizing the pathogenesis of African-lineage ZIKV compared to Asian-lineage ZIKV but did not seek to understand the mechanisms of the vertical transmission of ZIKV or the potential mechanisms underlying differences between the lineages. Future studies should investigate these mechanisms and conduct more-thorough epidemiological studies of African-lineage ZIKV, which may shed light on the reasons why ZIKV had not been associated with fetal harm prior to the outbreak in the Americas.

## MATERIALS AND METHODS

### Experimental design.

This study was designed to assess the pathogenic potential of a low-passage-number African-lineage ZIKV isolate during pregnancy in a nonhuman-primate model. Four pregnant Indian-origin rhesus macaques (Macaca mulatta) were inoculated subcutaneously with 1 × 10^4^ PFU of ZIKV-DAK between 44 and 50 days of gestation (term is 165 ± 10 days). Macaques were monitored throughout the remainder of gestation. At approximately gestational day 155, infants were delivered via C-section and humanely euthanized. A comprehensive set of maternal biopsy specimens, maternal-fetal interface tissues, and fetal tissues were collected for analysis. For the Asian-lineage group, four pregnant Indian-origin rhesus macaques (Macaca mulatta) were inoculated subcutaneously with 1 × 10^4^ PFU of ZIKV-PR between 44 and 50 days of gestation (term is 165 ± 10 days). Macaques were monitored throughout the remainder of gestation. At approximately gestational day 160, infants were delivered via cesarean section and were monitored for long-term development. A comprehensive set of maternal biopsy specimens and maternal-fetal interface tissues was collected for analysis. A cohort of four pregnant phosphate-buffered saline (PBS)-inoculated animals served as a control group and underwent the same experimental regimen, including the sedation for all blood draws and ultrasounds, as the ZIKV-PR cohort. Demographic data on the dams from each cohort are given in Table 4 below.

**TABLE 4:** Macaque demographics

Cohort	Animal ID^*a*^	Age at time of inoculation (yrs)	Gravidity (n)	Parity (n)
ZIKV-DAK	030-101	10.8	3	3
	030-102	19.0	7	7
	030-103	13.4	6	6
	030-104	15.2	10	10^*b*^
	Avg	14.6	6.5	6.5
ZIKV-PR	044-101	16.4	6	6
	044-102	17.1	7	7
	044-103	13.1	3	3
	044-104	7.6	2	2
	Avg	13.6	4.5	4.5
Mock	044-105	11.4	3	3
	044-106	15.4	6	6
	044-107	8.6	4	4
	044-108	12.5	5	5
	Avg	12.0	4.5	4.5

a All animals studied were female.

b Eight pregnancies took place at another facility before the animals’ arrival at WNPRC. The outcomes of these pregnancies are unknown, but they are assumed to have gone to term.

### Ethical approval.

This study was approved by the University of Wisconsin College of Letters and Sciences and Vice Chancellor for Research and Graduate Education Centers Institutional Animal Care and Use Committee (protocol numbers G005401 and G006139).

### Care and use of macaques.

All macaque monkeys used in this study were cared for by the staff at the Wisconsin National Primate Research Center (WNPRC) in accordance with the regulations and guidelines outlined in the Animal Welfare Act, the *Guide for the Care and Use of Laboratory Animals* (36), and the recommendations of the Weatherall report (https://royalsociety.org/topics-policy/publications/2006/weatherall-report/). All macaques used in the study were free of *Macacine herpesvirus 1*, simian retrovirus type D (SRV), simian T-lymphotropic virus type 1 (STLV), and simian immunodeficiency virus. For all procedures (including physical examinations, virus inoculations, ultrasound examinations, and blood collection), animals were anesthetized with an intramuscular dose of ketamine (10 mg/kg). Blood samples were obtained using a Vacutainer system or needle and syringe from the femoral or saphenous vein.

### Cells and viruses.

ZIKV/Aedes africanus/SEN/DAK-AR-41524/1984 (ZIKV-DAK) was originally isolated from Aedes africanus mosquitoes with a round of amplification on Aedes pseudocutellaris cells, followed by amplification on C6/36 cells and two rounds of amplification on Vero cells. ZIKV-DAK was obtained from BEI Resources (Manassas, VA). Zika virus/H.sapiens-tc/PUR/2015/PRVABC59_v3c2 (ZIKV-PR) was originally isolated from a human in Puerto Rico in 2015, with three rounds of amplification on Vero cells, and was obtained from Brandy Russell (CDC, Fort Collins, CO, USA). African green monkey kidney cells (Vero cells; ATCC CCL-81) were maintained in Dulbecco’s modified Eagle medium (DMEM) supplemented with 10% fetal bovine serum (FBS; HyClone, Logan, UT), 2 mM l-glutamine, 1.5 g/liter sodium bicarbonate, 100 U/ml penicillin, and 100 μg/ml of streptomycin and were incubated at 37°C under 5% CO_2_. Aedes albopictus mosquito cells (C6/36; ATCC CRL-1660) were maintained in DMEM supplemented with 10% FBS (HyClone, Logan, UT), 2 mM l-glutamine, 1.5 g/liter sodium bicarbonate, 100 U/ml penicillin, and 100 μg/ml of streptomycin and were incubated at 28°C under 5% CO_2_. The cell lines were obtained from the American Type Culture Collection, were not further authenticated, and were not specifically tested for mycoplasma. Virus stocks were prepared by inoculation onto a confluent monolayer of C6/36 cells; a single, clarified stock was harvested for each virus, with titers of 7.3 × 10^8^ PFU/ml for ZIKV-DAK and 1.58 × 10^7^ PFU/ml for ZIKV-PR. Deep sequencing with limited PCR cycles confirmed that the ZIVK-DAK stock was identical to the reported sequence in GenBank (accession number KY348860) at the consensus level. Five nucleotide variants were detected at frequencies of 5.4 to 13.1% (Table 5). PCR-free deep sequencing did not detect any evidence of Dezidougou virus, an insect-specific negevirus that is present in some ZIKV-DAK stocks. Amplicon deep sequencing of the ZIKV-PR stock using the methods described by Quick et al. (37) revealed two consensus-level nucleotide substitutions in the stock compared to the reported sequence in GenBank (accession number KU501215), as well as seven other minor nucleotide variants detected at frequencies of 5.3 to 30.6% (Table 5).

**TABLE 5:** Variants in challenge stocks

Challenge stock (GenBank accession no.)	Mutation	Nucleotide position (gene)	Frequency (%)	Amino acid change
ZIKV/Aedes africanus/SEN/DAK-AR-41524/1984 (KY348860)	Deletion (–A)	206 (capsid)	5.4	Frameshift
	T→G	211 (capsid)	5.9	I→S
	T→C	470 (Pr)	13.1	None
	C→T	3790 (NS2A)	9.1	A→V
	CGC→GCG	10311 (NS5)	5.7–5.9	R→A
Zika virus/H.sapiens-tc/PUR/2015/PRVABC59_v3c2 (KU501215)	Insertion (+A)	118 (capsid)	7.0	Frameshift
	G→T	1964 (envelope)	93.0	V→L
	T→G	2780 (NS1)	5.5	W→G
	T→C	3147 (NS1)	9.1	M→T
	C→T	5679 (NS3)	65.5	S→F
	C→T	7915 (NS5)	9.1	None
	T→C	9238 (NS5)	5.3	None
	G→A	9343 (NS5)	30.6	None
	Insertion (+C)	10446 (noncoding)	6.5	N/A

### Plaque assay.

All titrations for virus quantification from virus stocks and screens for infectious ZIKV from macaque tissue were completed by plaque assay on Vero cell cultures as described previously (38). Briefly, duplicate wells were infected with 0.1-ml aliquots from serial 10-fold dilutions in growth medium, and virus was adsorbed for 1 h. Following incubation, the inoculum was removed, and monolayers were overlaid with 3 ml containing a 1:1 mixture of 1.2% Oxoid agar and 2× DMEM (Gibco, Carlsbad, CA) with 10% (vol/vol) FBS and 2% (vol/vol) penicillin-streptomycin (100 U/ml penicillin, 100 μg/ml of streptomycin). Cells were incubated at 37°C under 5% CO_2_ for 4 days for plaque development. Cell monolayers were then stained with 3 ml of an overlay containing a 1:1 mixture of 1.2% Oxoid agar and 2× DMEM with 2% (vol/vol) FBS, 2% (vol/vol) penicillin-streptomycin, and 0.33% neutral red (Gibco). Cells were incubated overnight at 37°C, and plaques were counted.

### Inoculations.

Inocula were prepared from the viral stocks described above. The stocks were thawed, diluted in PBS to 10^4^ PFU/ml, and loaded into a 1-ml syringe that was kept on ice until challenge. Animals were anesthetized as described above, and 1 ml of the inoculum was delivered subcutaneously over the cranial dorsum. Animals were monitored closely following inoculation for any signs of an adverse reaction.

### Ultrasound measurements.

Ultrasound measurements were taken according to the procedures described previously (21). Briefly, dams were sedated with ketamine hydrochloride (10 mg/kg) for weekly sonographic assessment to monitor the health of the fetus (heart rate) and to take fetal growth measurements, including the fetal femur length (FL), biparietal diameter (BPD), head circumference (HC), and abdominal circumference (AC). Weekly fetal measurements were plotted against mean measurement values and standard deviations for fetal macaques developed at the California National Primate Research Center (27, 28). Additional Doppler ultrasounds were taken as requested by veterinary staff.

Gestational age standardized growth parameters for fetal HC, BPD, AC, and FL were evaluated by calculating gestational-age-specific Z-values from normative fetal growth parameters using data obtained from the California National Primate Research Center. A Z-score of 0, for example, corresponds to the median of the reference population, while a Z-score of 1.64 corresponds to the upper 95th percentile. Linear mixed-effects modeling with animal-specific random effects was used to analyze the fetal growth trajectories with advancing gestational age. In order to account for differences in fetal growth parameters at the date of inoculation, changes in fetal growth parameters from the date of inoculation (∼day 50) were analyzed. That is, changes in fetal growth parameters from the date of inoculation were regressed on gestational age (in weeks). An autoregressive correlation structure was used to account for correlations between repeated measurements of growth parameters over time. The growth trajectories were quantified by calculating the regression slope parameters, which were reported along with the corresponding 95% confidence intervals (CI). Fetal growth was evaluated both within and between groups. All reported *P* values are two-sided, and a *P* value of < 0.05 was used to define statistical significance. Statistical analyses were conducted using SAS software (SAS Institute, Cary, NC), version 9.4.

### Viral RNA isolation from blood.

Viral RNA was isolated from macaque blood samples as described previously (20, 38). Briefly, plasma was isolated from EDTA-anticoagulated whole blood on the day of collection using Ficoll density centrifugation for 30 min at 1,860 × *g* if the blood was being processed for peripheral blood mononuclear cells (PBMC), or it was centrifuged in the blood tube at 1,400 × *g* for 15 min. The plasma layer was removed, transferred to a sterile 15-ml conical tube, and spun at 670 × *g* for an additional 8 min to remove any remaining cells. Viral RNA was extracted from a 300-μl plasma aliquot using the Viral Total Nucleic Acid kit (Promega, Madison, WI) on a Maxwell 16 MDx or Maxwell RSC 48 instrument (Promega, Madison, WI).

### Viral RNA isolation from tissues.

Tissue samples, cut to a 0.5-cm thickness on at least one side, were stored in RNAlater at 4°C for 2 to 7 days. RNA was recovered from tissue samples using a modification of the method described by Hansen et al. in 2013 (39). Briefly, up to 200 mg of tissue was disrupted in TRIzol (Life Technologies) with two 5-mm-diameter stainless steel beads using the TissueLyser (Qiagen) for 3 min at 25 rps twice. Following homogenization, samples in TRIzol were separated using bromo chloropropane (Sigma). The aqueous phase was collected, and glycogen was added as a carrier. The samples were washed in isopropanol and ethanol precipitated. RNA was fully resuspended in 5 mM Tris (pH 8.0).

### QRT-PCR.

vRNA isolated from both fluid and tissue samples was quantified by quantitative reverse transcription-PCR (QRT-PCR) as described previously (11). The RT-PCR was performed using the SuperScript III Platinum One-Step Quantitative RT-PCR system (Invitrogen, Carlsbad, CA) on a LightCycler 96 or LightCycler 480 instrument (Roche Diagnostics, Indianapolis, IN). The viral RNA concentration was determined by interpolation onto an internal standard curve composed of seven 10-fold serial dilutions of a synthetic ZIKV RNA fragment based on a ZIKV strain derived from French Polynesia that shares >99% identity at the nucleotide level with the Puerto Rican strain used in the infections described in this report. Each viral load reported here is the average of two replicates.

### Statistical analysis of viral loads.

Plasma viral load curves were generated using GraphPad Prism software. The area under the curve of 0 to 10 dpi was calculated, and a two-sample *t* test was performed to assess differences in the peak, duration, and area under the curve of viremia between macaques infected with ZIKV-DAK and those infected with ZIKV-PR. To compare differences in the viral burden in the maternal-fetal interface, a nonparametric Mann-Whitney test was used to assess differences in each of the maternal-fetal interface tissues. GraphPad Prism 8 software was used for these analyses.

### PRNT.

Macaque serum was isolated from whole blood on the same day it was collected by using a serum separator tube (SST). The SST was centrifuged for at least 20 min at 1,400 × *g*, and the serum layer was removed, placed in a 15-ml conical tube, and centrifuged for 8 min at 670 × *g* to remove any additional cells. Serum was screened for ZIKV neutralizing antibodies by utilizing a plaque reduction neutralization test (PRNT) on Vero cells as described in reference 40 against ZIKV-PR and ZIKV-DAK. The neutralization assay was performed with the same virus stock that was used for the challenge: ZIKV-DAK was used to test samples from the African-lineage cohort, and ZIKV-PR was used to test samples from the Asian-lineage cohort. Neutralization curves were generated using GraphPad Prism 8 software. The resulting data were analyzed by nonlinear regression to estimate the dilution of serum required to inhibit 50% and 90% of infection. The data shown in Fig. 3 reflect a single experiment.

### ZIKV IgM ELISA.

A commercial ZIKV IgM ELISA kit (EI 2668–9601 M; Euroimmun, Lübeck, Germany) was used to determine if IgM antibodies were present in fetal serum samples at the time of delivery. The protocol was followed as specified by the manufacturer, and all samples were frozen undiluted at −80°C until use. Duplicates were run for each of the samples. In addition to the positive and negative controls included with the kit, a 14-dpi serum sample from macaque 030-104 was used as a positive control and a 4-dpi serum sample from 030-104 was used as a negative control. All serum samples were diluted 1:100. Immediately upon addition of the stop solution, the plate was read at 450 nm. An extinction value was calculated for each sample by calculating the ratio of the optical density (OD) of the sample to the OD of the assay calibration sample (Fig. 5C). Per the kit’s recommendation, any sample with a >1.1 ratio was considered to be positive for ZIKV IgM, between 1.1 and 0.8 was considered to be borderline, and <0.8 was considered to be negative.

### Cesarean section and tissue collection.

Between 155 and 160 days of gestation, infants were delivered via cesarean section, and tissues were collected. The fetus, placenta, fetal membranes, umbilical cord, and amniotic fluid were collected at surgical uterotomy, and maternal tissues were biopsied during laparotomy. These were survival surgeries for the dams. Fetuses born to dams infected with ZIKV-DAK were euthanized with an overdose of sodium pentobarbital (50 mg/kg), and the entire conceptus (fetus, placenta, fetal membranes, umbilical cord, and amniotic fluid) was collected and submitted for tissue collection and necropsy. For fetuses born to dams infected with ZIKV-PR, the infant was removed from the amniotic sac, the umbilical cord clamped, and neonatal resuscitation performed as needed. The placenta, amniotic fluid, and fetal membranes were then collected. Infants were placed with their mothers following the dams’ recovery from surgery.

Tissues were dissected as described previously (21) using sterile instruments that were changed between each organ and tissue type to minimize possible cross contamination. Each organ/tissue was evaluated grossly *in situ*, removed with sterile instruments, placed in a sterile culture dish, and sectioned for histology and viral burden assay and/or banked for future assays. The sampling priority for small or limited fetal tissue volumes (e.g., thyroid gland, eyes) was vRNA followed by histopathology, so not all tissues were available for both analyses. A comprehensive listing of all specific tissues collected and analyzed is presented in Fig. 5A (maternal-fetal interface tissues) and Table 1 (maternal biopsy specimens and fetal tissues). Biopsy specimens of the placental bed (uterine placental attachment site containing deep decidua basalis and myometrium), maternal liver, spleen, and a mesenteric lymph node were collected aseptically during surgery into sterile petri dishes, weighed, and further processed for viral burden and, when a sufficient sample size was obtained, histology.

In order to more accurately capture the distribution of ZIKV in the placenta, each placental disc was separated, and fetal membranes were sharply dissected from the margin, weighed, measured, and placed in a sterile dish on ice. A 1-cm-wide cross section was taken from the center of each disc, including the umbilical cord insertion on the primary disc, and placed in 4% paraformaldehyde (PFA). Individual cotyledons, or perfusion domains, were dissected using a scalpel and were placed in individual petri dishes. From each cotyledon, a thin center cut was taken using a razor blade and was placed in a cassette in 4% paraformaldehyde. Once the center cut was collected, the decidua and the chorionic plate were removed from the remaining placenta. From each cotyledon, pieces of decidua, chorionic plate, and chorionic villi were collected into two different tubes with different media for vRNA isolation and for other virological assays.

### Histology.

Following collection, tissues were handled as described previously (38). All tissues (except neural tissues) were fixed in 4% paraformaldehyde for 24 h and were transferred into 70% ethanol until processing and embedding in paraffin. Neural tissues were fixed in 10% neutral buffered formalin for 14 days until processing and embedding in paraffin. Paraffin sections (5 μm for all tissues other than the brain [sectioned at 8 μm]) were stained with hematoxylin and eosin (H&E). Pathologists were blinded to vRNA findings when tissue sections were evaluated microscopically. Photomicrographs were obtained using bright-light Olympus BX43 and Olympus BX46 microscopes (Olympus Inc., Center Valley, PA) with an attached Olympus DP72 digital camera (Olympus Inc.) and Spot Flex 152 64 Mp camera (Spot Imaging) and were captured using commercially available image analysis software (cellSens Dimension [Olympus Inc.] and Spot software, version 5.2).

### Placental histology scoring.

Pathological evaluations of the cross sections of each of the placental cotyledons were performed by individuals blinded to the experimental condition. Each of the cross sections was evaluated for the presence of chronic histiocytic intervillositis (CHIV), infarctions, villous stromal calcifications, and vasculopathy. Three-way analysis of variance (ANOVA) was performed to assess statistical significance among groups for each parameter, including placental weight.

Two of three boarded pathologists, blinded to vRNA findings, independently reviewed the central cross section of each placental disc and quantitatively scored the placentas on 22 independent criteria. Six of these are general criteria assessing placental function, 2 assess villitis, 3 assess the presence of fetal malperfusion, and 11 assess the presence of maternal malperfusion. Once initial scores were assigned, pathologists met to discuss and resolve any significant discrepancies in scoring. Scores were assigned to each placental disc for most parameters unless the evaluation score corresponded to the function of the entire placenta.

For criteria that were measured on a quantitative scale, median scores and interquartile ranges were calculated for each experimental group. For criteria that were measured on a binary “present/not present” scale, the cumulative incidence in each experimental group was calculated as a frequency and a percentage. For quantitative criteria, a nonparametric Wilcoxon rank test was used to calculate the statistical significance of differences between each of the groups and between the mock-inoculated group and the two ZIKV-infected groups. For binary features, Fisher’s exact test was used to calculate the statistical significance of differences between each of the groups and between the mock-inoculated group and the two ZIKV-infected groups. To determine whether chronic villitis correlated with the criteria assessing fetal malperfusion and whether chronic deciduitis correlated with the criteria assessing maternal malperfusion, scores were adjusted to be on the same scale (i.e., converting measures on a 0-to-1 scale to a 0-to-2 scale) so that each parameter carried equal weight in the combined score. A nonparametric Spearman correlation was used to determine the correlation.

### *In situ* hybridization.

*In situ* hybridization was conducted on cross sections of placental cotyledons as described previously (22). Briefly, tissues were fixed in 4% PFA, alcohol processed, and paraffin embedded. Commercial ISH probes against the ZIKV genome (catalog no. 468361; Advanced Cell Diagnostics, Newark, CA, USA) were used. ISH was performed using the RNAscope Red 2.5 kit (catalog no. 322350; Advanced Cell Diagnostics) according to the manufacturer's instructions.

### Data availability.

All of the data used for figure generation and statistical analysis in this study can also be found at https://github.com/cmc0043/african-lineage-zikv-in-pregnant-macaques. In the future, primary data that support the findings of this study will also be available at the Zika Open Research Portal (https://openresearch.labkey.com/project/ZEST/begin.view). Data for the ZIKV-DAK-infected cohort can be found under study ZIKV-030; data for ZIKV-PR and mock-inoculated cohorts can be found under ZIKV-044. Raw FASTQ reads (BioProject accession number PRJNA673500) and a FASTA consensus sequence (BioProject accession number PRJNA476611) of the challenge stock of ZIKV/Aedes africanus/SEN/DAK-AR-41524/1984 are available at the Sequence Read Archive. Raw FASTQ reads of the challenge stock of ZIKV PRVABC59 are available at the Sequence Read Archive under BioProject accession number PRJNA392686. We declare that all other data supporting the findings of this study are available within the article.
